# Exploring factors associated with bleeding events after open heart surgery in patients on dialysis − effects of the presence or absence of warfarin therapy

**DOI:** 10.1186/s40780-024-00353-x

**Published:** 2024-07-12

**Authors:** Masanori Suzuki, Yuki Hasegawa, Hiroaki Tanabe, Masayoshi Koinuma, Ryohkan Funakoshi

**Affiliations:** 1 Faculty of Pharmaceutical Sciences, Teikyo Heisei University, Tokyo, Japan; 2 Department of Pharmacy, Kameda Medical Center, Chiba, Japan; 3 Department of Cardiovascular surgery, Kameda Medical Center, Chiba, Japan; 4 Department of Pharmacy Administration, Kameda Medical Center, Chiba, Japan

**Keywords:** Valve replacement, Valvuloplasty, Warfarin therapy, Dialysis, Perioperative management

## Abstract

**Background:**

Perioperative management of patients on dialysis is critical for controlling bleeding and thrombotic risk, in addition to infection control. Postoperative anticoagulation is often difficult to control, and different institutions have different policies. Therefore, in this study, we aimed to investigate factors associated with postoperative bleeding events and whether warfarin (WF) therapy affects the incidence of postoperative bleeding events, total mortality, and stroke.

**Methods:**

Patients who were admitted to the cardiovascular surgery department and underwent valve replacement or plasty were included, and those who underwent mechanical valve introduction were excluded. Thirty-nine patients were included in the study. The primary endpoint was to identify factors associated with the composite endpoint of postoperative bleeding events, and the secondary endpoint was to determine the effect size of WF therapy on postoperative bleeding events, all-cause mortality, and stroke and the strength of association between the crossed endpoints. The strength of the association between the crossed items was examined.

**Results:**

Low body weight (*p* = 0.038) was identified as a factor associated with the primary endpoint of postoperative bleeding events. The secondary endpoint of whether or not patients received WF therapy was largely unrelated to bleeding events, all-cause mortality, and postoperative stroke up to 90 days after surgery.

**Conclusions:**

Preliminary studies suggest that low body weight is a risk factor for postoperative bleeding events in patients on dialysis, although further exploration of other factors will be necessary with the accumulation of similar cases.

## Background

The number of patients with diabetes mellitus, a lifestyle-related disease, and those on dialysis owing to diabetic nephropathy is increasing. The leading causes of death among patients on dialysis are infections and cardiovascular diseases [1–3]. Furthermore, it has been reported that vascular calcification continues to progress in patients even after the initiation of dialysis [4] and that it has a concomitant effect on valvular heart disease [5, 6]. Hence, perioperative management of patients on dialysis undergoing cardiovascular surgeries requires additional attention owing to the risks of cerebral hemorrhage and gastrointestinal bleeding [7]. In 2011, guidelines [8] for the evaluation and treatment of cardiovascular complications in patients on hemodialysis were developed, and addressing cardiovascular disorders in these patients is paramount. Atrial fibrillation (AF) is one of the most common arrhythmias that can occur after cardiovascular surgery [9–11]. Anticoagulation should be introduced as soon as possible owing to the risk of thromboembolism and stroke in all cases [12]. This is also applicable to patients on dialysis. In Japan, warfarin (WF) is contraindicated in patients on dialysis in principle. However, its administration should be strictly controlled and only considered for patients who would benefit from treatment [8]. In general, chronic kidney disease and dialysis are risk factors for bleeding [13], and other factors include low body weight and age [14, 15]. In the perioperative period, WF should be used as a standard anticoagulation therapy after valve replacement or valvuloplasty for thromboprophylaxis while considering the risk of bleeding [12]. A meta-analysis has indicated that WF increases the risk of hemorrhagic stroke and major bleeding without reducing the risk of stroke or total mortality [16]. Notably, there is a lack of information on the availability of WF therapy in the management of postoperative valve replacement and valvuloplasty in the field of cardiovascular surgery. WF therapy was used for short periods owing to the risk of postoperative bleeding. Therefore, in this study, we aimed to explore factors associated with postoperative bleeding events in patients on dialysis and to determine the influence of the introduction of WF therapy.

## Methods

### Study design

This was a retrospective, observational, single-center study conducted in accordance with the tenets of the Declaration of Helsinki. Patient consent was obtained through an opt-out approach.

### Patients enrolled

The study included patients on dialysis who were aged ≥ 20 years and underwent valve replacement or valvuloplasty after admission to the Division of Cardiovascular Surgery at Kameda General Hospital between June 1, 2015, and December 31, 2020. Patients with mechanical valves were excluded. Thirty-nine patients were included in the study.

### Variables

For each item, data were obtained from Kameda Medical Center’s electronic medical records.

#### Primary endpoints

The number of days to postoperative bleeding events was defined as a composite endpoint. The smaller number of days from the date of surgery to the date of re-thoracic surgery owing to bleeding or the date of drop in hemoglobin level ≥ 20 g/L was used as the difference from the previous day as the endpoint. Cases where re-thoracotomy was performed immediately after the surgery (on the same day or next day) were not included in the event analysis to exclude bleeding directly attributable to the surgery. Further subgroup analysis was performed to determine whether blood transfusion had any effect.

#### Secondary endpoints

Secondary endpoints included WF therapy induction and death up to 90 days after surgery and postoperative stroke.

#### Participant attributes

During each observation period, the following data were collected: dialysis types (hemo and peritoneal), dialysis history, age, sex, height, weight, body mass index, preoperative Hb level, New York Heart Association classification, preoperative risk assessment (Euro-Score II), AF, beta-blocker use, WF use, anti-platelet drug use, and details of the operation (aortic valve replacement).

#### Items related to the participants’ intraoperative and postoperative course

The following data were obtained from the electronic medical records: operative time, intraoperative blood loss, cardiopulmonary duration, aortic blocking time, length of hospital stay, and transfer to another department.

### Statistical analysis

#### Summary statistics

Summary statistics were calculated for the primary endpoints, secondary endpoints, participant demographics, and items related to participants’ intraoperative and postoperative course.

#### Analyzing primary endpoints

The observation period was until the 90th postoperative day. In one case, Euro-score II could not be confirmed, and it was considered a missing value.

To screen for explanatory variables related to the primary endpoints, analyses were conducted with the primary endpoints as the objective variables and participant attributes as explanatory variables. The statistical significance level was set at *p* < 0.15 for convenience, as the purpose of the analysis was screening. Histograms of individual explanatory variables were checked, and logarithmic transformations were used to approximate normal distribution in cases where the distribution was clearly abnormal to improve the fit to the regression model.

A multivariate Cox proportional hazards regression analysis was performed using the variables obtained here. To avoid multicollinearity, one of the variables was adopted from a medical perspective if the correlation coefficient between variables was greater than 0.7. Furthermore, an interaction term was added to the explanatory variables in addition to the main effect to clarify the relationships among the explanatory variables in the regression model (Model 1). Furthermore, a separate model was created wherein WF therapy was introduced as an explanatory variable even when the p-value was not < 0.1 in the univariate Cox proportional hazards regression analysis (Model 2). Corrected Akaike information criteria (AICc) and Bayesian information criteria (BIC) comparisons of Model 1 and Model 2 were also conducted to confirm the fit as a regression model. If the fit of Model 2 was at least better than that of Model 1, it was considered that the introduction of WF therapy might have affected the occurrence of the event.

#### Analyzing secondary endpoints

The effect size was calculated for the presence or absence of postoperative hemorrhagic events, death, and postoperative stroke up to 90 days postoperatively, with or without WF therapy, and the strength of the association was examined between the crossed items. The type of effect size was determined as Cramer’s V [17] and Yule’s Q [18], and the magnitude of the difference was determined based on each criterion. Cramer’s V values range between 0 and 1, with 0 indicating no association at all between variables and 1 indicating a perfect association. On the contrary, Yule’s Q values range from − 1 to + 1, where − 1 means a complete negative association, + 1 means a complete positive association, and 0 indicates no association.

#### Analysis software

JMP ver. 17.2 was used for statistical analysis, and the statistical significance level was set to below 5%.

## Results

Thirty-nine participants were included in the study, and their summary statistics were calculated (Tables 1, 2 and 3). For the primary endpoint, only the data of the date of drop in hemoglobin level ≥ 20 g/L were used. In the analysis of the primary endpoint, explanatory variables were screened, and age, weight, and body mass index (BMI) were extracted as variables. Although none of these variables were statistically significant, the results suggested that age increased the risk of an event by an average of 10% for each additional year of age (hazard ratio [HR] = 1.100, 95% confidence interval [CI]: 0.974–1.267, *p* = 0.136). Body weight possibly decreased the risk of events by an average of 7.7% for each kilogram gained (HR = 0.923, 95% CI: 0.837–1.001, *p* = 0.054). As for BMI, the results suggested an average 24.1% reduction in the risk of events per unit increase (HR = 0.759, 95% CI: 0.500–1.015, *p* = 0.067). Additionally, maintenance dialysis type and AF were excluded because the calculation results did not converge, and estimates could not be calculated (Table 4).

**Table 1:** Summary statistics of the participants (*n* = 39)

Characteristic	Mean [SD] or *n* (%)	
Age (years)	71.0	[8.7]
Male	30	(76.9)
Hemodialysis	37	(94.9)
CAPD	4	(10.3)
Height (cm)	159.1	[9.3]
Body weight (kg)	59.8	[16.6]
Body mass index (kg/m^2^)	23.4	[4.7]
Hb (g/dL)	11.4	[1.5]
AF	8	(20.5)
Beta-blocker	19	(48.7)
Anti platelet	31	(79.5)
Euro-score II	6.7	[6.7]
NYHA	-	-
1	6	(15.4)
2	24	(61.5)
3	6	(15.4)
4	3	(7.7)
EF (%)	57.7	[15.9]

AF, atrial fibrillation; CAPD, continuous ambulatory peritoneal dialysis;

EF, ejection fraction, NYHA, New York Heart Association; SD, standard deviation

**Table 2:** Summary statistics of procedure and postoperative data (*n* = 39)

	Mean [SD] or *n* (%)	
Elective	34	(87.2)
Urgent	4	(10.3)
Emergent	1	(2.6)
Procedure	-	-
AVR	28	(71.8)
MVR	2	(5.1)
AVP	11	(28.2)
MVP	10	(25.6)
TVP	1	(2.6)
MAP	1	(2.6)
TAP	8	(20.5)
CABG	12	(30.8)
MAZE	4	(10.3)
Left atrial appendage closure, suture, resection	16	(41.0)
Operating time (min)	379.4	[121.2]
Blood loss (mL)	223.3	[184.2]
Cardiopulmonary bypass time (min)	244.7	[101.6]
Aortic cross-clamping time (min)	152.9	[83.4]
Transfer department	9	(23.1)
Length of hospital stay (days)	38.6	[39.8]
Blood transfusion	38	(97.4)

AVR, aortic valve replacement; MVR, mitral valve replacement; AVP, aortic valve plasty; MVP, mitral valve plasty; TVP, tricuspid valvuloplasty; MAP, mitral annuloplasty; TAP, tricuspid annuloplasty; CABG, coronary artery bypass graft

**Table 3:** Summary statistics of primary and secondary outcomes

	Mean [SD] or *n* (%)	
Postoperative days until Hb drop	80.9	[24.8]
WF therapy	18	(46.2)
Total mortality	6	(15.4)
Stroke	0	0

Hb, hemoglobin; SD, standard deviation; WF, warfarin

**Table 4:** Univariate Cox proportional hazards regression analysis

	HR	Lower 95% CI	Upper 95% CI	*p*
Age	1.100	0.974	1.267	0.136^§^
Male	0.437	0.073	2.617	0.365
Height	0.946	0.863	1.041	0.384
BW	0.923	0.837	1.001	0.054^§^
BMI	0.759	0.500	1.015	0.067^§^
Hb	0.832	0.416	1.557	0.245
NYHA	1.131	0.326	3.222	0.832
EF	1.029	0.969	1.127	0.392
Euro-score II※	1.964	0.619	6.354	0.245
Beta-blocker	0.696	0.116	4.164	0.691
WF	0.758	0.127	4.540	0.762
Anti-platelet	0.358	0.060	2.148	0.261
AVR	1.684	0.188	15.080	0.641

^§^Because the purpose of the analysis was screening, the statistical significance level was set at *p* < 0.15 for convenience, and items were selected for multivariate analysis

※natural logarithmic transformation. AVR, aortic valve replacement; BMI, body mass index; BW, body weight; CI, confidence interval; EF, ejection fraction; Hb, hemoglobin; HR, hazard ratio; NYHA, New York Heart Association

The estimated correlation coefficients between age, body weight, and BMI showed a strong positive correlation between body weight and BMI (*r* = 0.8843); therefore, we decided to remove the BMI side from the screening and used age, body weight, and their interaction terms (age × body weight) in the multivariate Cox proportional hazards regression analysis.

The results of Model 1 showed that body weight had a significant effect on bleeding events after open heart surgery (*p* = 0.0453). Specifically, each 1 kg increase in body weight was associated with an average potential 11% reduction in bleeding events after open heart surgery (HR = 0.893, 95% CI: 0.778–0.9981, *p* = 0.0453). Contrarily, age showed a trend toward a hazard ratio of 1.18 for each additional year of age, although it did not reach statistical significance (*p* = 0.115).

The interaction term between body weight and age (BW × Age) did not significantly contribute to the model (*p* = 0.3731).

In Model 2, the analysis was performed by forcing the explanatory variables used in Model 1 with and without the introduction of WF therapy; age and body weight affected the HR for Hb level drop, as in Model 1. For body weight, the hazard of Hb level drop was estimated to be 0.882 times greater for each 1 kg decrease, and this was statistically significant (*p* = 0.038). For age, a 1.185-fold increase in the hazard of bleeding events for each additional year of age was suggested, but it was not statistically significant (*p* = 0.103). Additionally, body weight and the age interaction term (BW × Age) (*p* = 0.489) and WF use (*p* = 0.582) showed results that were not statistically significant.

Model 1 showed lower values for AICc and BIC than Model 2 did, suggesting that Model 1 is a better fit for the data. We concluded that the effect of WF therapy on the occurrence of events was insignificant, as Model 1 was a better fit than Model 2 (Table 5).

**Table 5:** Multivariate Cox proportional hazards regression analysis

	Item	EV	SE	HR	Lower 95% CI	Upper 95% CI	Reci-procal	*p*		AICc	BIC	*p*
Model 1 Age + BW + (Age × BW)	Age	0.167	0.114	1.182	0.967	1.531	0.846	0.115		36.590	40.895	0.100
	BW	-0.113	0.063	0.893	0.778	0.998	1.120	0.045				
	(BW-59.762 kg) × (Age-70.974 years)	0.008	0.009					0.373				
Model 2 Age + BW + (Age × BW) +WF	Age	0.169	0.113	1.185	0.972	1.530	0.844	0.103		38.778	44.256	0.162
	BW	-0.125	0.070	0.882	0.754	0.994	1.133	0.038				
	(BW-59.762 kg) × (Age-70.974 years)	0.006	0.009					0.489				
	WF	0.324	0.588	1.910	0.191	19.119		0.582				

AICc, corrected Akaike information criteria; BIC, Bayesian information criteria; BW, body weight; CI, confidence interval; EV, estimated value; HR, hazard ratio; SE, standard error; WF, warfarin

Since there was only one case in the no blood transfusion group, the analysis was conducted using data from the blood transfusion group. The results showed that the weight factor in Model 2 was slightly below the significance level (*p* = 0.0692) but had almost no effect on the partial regression coefficient values.

During the 90-day postoperative period, 11.11% (two patients) of patients in the WF therapy group had a bleeding event, and 14.29% (three patients) of patients in the non-WF therapy group had a bleeding event (Table 6). The strength of association for the presence of bleeding events was 0.047 for Cramer’s V and 0.143 for Yule’s Q. These values indicate negligible association between WF therapy and survival status.

**Table 6:** Effect size and strength of association between two crossed items

		Bleeding events (%) -	Bleeding events (%) +	Effect size	
WF therapy	+	16 (88.9)	2 (11.1)	Cramer’s V (0 to 1)	Yule’s Q (-1 to + 1)
	-	18 (85.7)	3 (14.3)	0.047	0.143
		Alive	Death		
	+	15 (83.3)	3 (16.7)	Cramer’s V (0 to 1)	Yule’s Q (-1 to + 1)
	-	18 (85.7)	3 (14.3)	0.033	-0.091
		Stroke (%) -	Stroke (%) +		
	+	18 (100)	0 (0)	Incomputable	
	-	21(100)	0 (0)		

SE, standard error; WF, warfarin

Regarding 90-day survival, 16.67% (three patients) of patients died in the WF therapy group, and 14.29% (three patients) died in the non-WF therapy group. The strength of the association for survival status was 0.033 for Cramer’s V and − 0.091 for Yule’s Q. These values indicate a negligible association between WF therapy and survival status. Contrarily, no stroke occurred in either group. Therefore, the association between WF therapy and stroke occurrence was not assessed. No cases of stroke were observed in either group; however, one patient in the non-WF therapy group experienced postoperative spinal cord infarction. This patient, who had multiple asymptomatic cerebral infarcts preoperatively, did not show improvement in the state of consciousness after surgery for infective endocarditis. Although the patient had hematochezia, thrombocytopenia, and frequent blood transfusions, the prothrombin time-international normalized ratio (PT-INR) and activated partial thromboplastin time remained within the target range for patients on hemodialysis. Therefore, the patient was unlikely to be affected by WF therapy.

## Discussion

Age, body weight, and BMI were extracted as factors for Hb level drop in the primary endpoint. Statistical analysis confirmed the effect of the interaction between body weight and age, although the interaction term was not significant, and weight was extracted as the most relevant factor. These factors have been reported as bleeding risk factors [19], and older adult patients may lose weight as they age [20]. Although the significance level was slightly lower in the group with blood transfusions, the partial regression coefficients were almost unaffected, suggesting that body weight was not the most influential factor. Furthermore, prolonged use of anticoagulants is a risk factor for bleeding [14] but was not detectable during the 90-day postoperative WF therapy period. A recent study [21] reported that there was a difference in bleeding events between direct oral anti-coagulants and WF at approximately 100 days after the start of use, suggesting that long-term use may influence bleeding events. These results suggest that body weight (rather than the presence or absence of WF use) is the most important contributor to postoperative bleeding events.

The secondary endpoints examined reflected the effect of WF therapy on postoperative bleeding events, all-cause mortality, and stroke. However, the opposite of the expected results was observed, with two cases in the WF therapy group and three cases in the non-WF therapy group meeting the criteria for bleeding events. Essentially, the introduction of WF therapy increases the likelihood of bleeding because of the inhibition of coagulation factors. However, in the WF group, only 1 of the 18 patients had a PT-INR of > 3, and no bleeding events requiring reopening of the chest were observed after the introduction of WF therapy, indicating that the patients could be safely managed. A previous study [16] reported an increased risk of bleeding. In this study, WF therapy was given for a limited period of 3 months postoperatively; therefore, there may have been no difference in postoperative bleeding events between patients who received and those who did not receive WF therapy. The fact that postoperative bleeding cases were observed even in the non-WF therapy group suggests that patients may have been prone to postoperative bleeding events owing to low body weight or other bleeding risk factors even without WF therapy. The induction of WF therapy may be less likely to increase the risk of postoperative bleeding events. In the factor analysis, being underweight was extracted as a factor to lower Hb levels. Being underweight is a bleeding risk factor [14], and it was considered to be more associated with Hb level drop than Euro-score II or age. We observed the death of patients on dialysis, regardless of their anticoagulation status. However, this observation could be attributed to the inclusion of high-risk patients (preoperative Euro-Score II 6.03% and 3.13%), that is, those undergoing emergency admissions. The in-hospital mortality rate for patients on dialysis who underwent cardiac surgery has been reported to be 12.7% [22]. A similar result of 4/39 cases (10.3%) in this study likely relates to the poor prognosis of patients on dialysis [23]. Patients on dialysis are at a high risk of infection and perioperative complications, as they are already anemic at baseline. It is reasonable to assume that the higher risk of perioperative complications and death in the WF therapy group, although not significantly different, may have been due to the high-risk situation of patients on dialysis rather than WF therapy. In addition, we encountered a patient who was scheduled for discharge but suddenly experienced a reversal in their condition, developing an infection during postoperative management. We found that this was not due to poor management of WF therapy or the onset of stroke caused by thrombosis, suggesting no relation between the patient outcome and the effect of WF therapy during the first 3 months after surgery. However, the emergence of postoperative embolism due to the decision not to introduce WF therapy must be avoided. If the policy is not to introduce WF after risk assessments of bleeding and thrombotic events, it is crucial to share the initial symptom and take measures to minimize the unlimited risk of embolic risk factors among healthcare professionals involved in postoperative monitoring.

In contrast, a significant increase in the length of hospital stay was observed in the group receiving WF therapy. We assumed that this was because of prolonged hospital stays due to bleeding control by WF or the time required to determine the optimal dose due to poor PT-INR control; however, as the actual hospital stay of more than 90 days was due to infection and transfer adjustment, it was not considered to be influenced by the presence or absence of WF therapy.

Preliminarily, this study suggests that caution is warranted in patients with low body weight as a risk for bleeding events in the perioperative management of cardiovascular surgery in dialysis patients.

This study had some limitations. First, we considered both scheduled and emergency surgeries. Therefore, it cannot be conclusively stated that the data represent real-world scenarios. Additionally, as this was a single-center, retrospective, observational study, no adjustments were made for potential confounding factors between groups. However, the number of dialysis patients undergoing cardiovascular surgery is small, and the implementation of postoperative anticoagulation therapy remains controversial. Therefore, as a preliminary study, we believe that the results of the factor analysis of this study can be used to guide future multicenter studies.

In conclusion, the preliminary findings of this study suggest that low body weight is a risk factor for postoperative bleeding events in patients on hemodialysis, but other factors should be explored in the future with the accumulation of similar cases.

## Data Availability

The datasets used and/or analyzed during the current study are available from the corresponding author on reasonable request.

## Abbreviations

AF: Atrial fibrillation
AICc: Corrected Akaike information criterion
AVP: Aortic valve plasty
BIC: Bayesian information criterion
BMI: Body mass index
BW: Body weight
CAPD: Continuous ambulatory peritoneal dialysis
CABG: Coronary artery bypass graft
CI: Confidence interval
DOAC: Direct oral anticoagulant
EF: Ejection fraction
Hb: Hemoglobin
HR: Hazard ratio
MAP: Mitral annuloplasty
MVP: Mitral valve plasty
MVR: Mitral valve replacement
NYHA: New York Heart Association
SD: Standard deviation
PT-INR: Prothrombin time-international normalized ratio
TAP: Tricuspid annuloplasty
TVP: Tricuspid valvuloplasty
WF: Warfarin
